# Exposure to Degludec During Pregnancy: A Case Report

**DOI:** 10.7759/cureus.5158

**Published:** 2019-07-17

**Authors:** Alejandro Roman-Gonzalez, Carlos A Builes-Barrera, Alejandra Aristizabal Baron

**Affiliations:** 1 Endocrinology, Universidad De Antioquia Hospital Universitario San Vicente Fundación, Medellin, COL; 2 Internal Medicine, Universidad De Antioquia, Medellin, COL

**Keywords:** pregnancy, insulin, degludec, glargine, detemir

## Abstract

A non-planned pregnancy in a patient with type 1 diabetes with ocular complications, was treated with degludec from pre-conception stages until the postpartum is reported. The newborn was healthy without any congenital abnormalities; however, due to respiratory distress required neonatal intensive care unit.

## Introduction

Pregnancy in women with type 1 diabetes might be a hazardous state, due to the difficulties concerning adequate metabolic control, which result in adverse outcomes for the mother and the fetus [1]. Known complications of diabetes during pregnancy have been described, including increased risk for hypoglycemia, preeclampsia, fetal macrosomia, and respiratory distress syndrome of the newborn, among other prenatal and perinatal complications [2]. The rationale for adequate glycemic control is clear; nevertheless, some patients have difficulties keeping up with glucose goals while on treatment with intermediate-acting neutral protamine Hagedorn (NPH) insulin or the long-acting insulin analogs such as detemir, which are approved for use during pregnancy. Although insulin degludec is not approved for use in pregnant women, this extra-long-acting insulin analog could be a potential alternative for those women with complicated glycemic control during pregnancy. However, the safety of insulin degludec during pregnancy is unknown and the reported data are limited to case reports. We report an unplanned pregnancy in a patient with type 1 diabetes with complications treated with degludec from preconception stages until postpartum.

## Case presentation

A 33-year-old patient with type 1 diabetes associated with high-risk proliferative retinopathy and A2 albuminuria (diagnosed at the age of 16) switched to insulin degludec two years prior to her last pregnancy. The patient was treated with insulin degludec (27 units) and fixed-dose insulin glulisine (10 units) with each meal. The patient became pregnant despite medical advice, the HbA1c during the first trimester was 6.1% (43 mmol/mol) without reported hypoglycemia. The medical team ordered her to change from degludec to insulin detemir; however, the patient was reluctant considering that insulin degludec provided better metabolic control compared to previous insulin regimens. Despite several explanations of the unknown risk of insulin degludec during pregnancy, the patient decided to continue the pregnancy with this insulin. The first trimester went through without complications, as well as the second trimester. The total insulin dose at the end of the third trimester was 30 IU of degludec daily and 18 IU of glulisine before each meal. Ultrasound at 34 weeks of pregnancy showed an anterior placenta, and the fetus presented in podalic version. The fetus’ weight was 2281 g (40th percentile). During week 35, the patient developed preeclampsia and underwent an emergency C-section. The child was born with a bodyweight of 2500 g (50th percentile), 43 cm in length (percentile 10-50th), had a low Apgar score at 1 min and 5 min (5/10 points), and required non-invasive ventilation in the neonatal intensive care unit for two days but did not present with any congenital defects. Currently, the baby is 10 months old and healthy.

## Discussion

Insulin degludec is not approved during pregnancy, and safety is currently unknown. The use of new insulin analogs may be associated with potential maternal or fetal complications due to their strength or to its potential binding to the IGF-1 receptor [3]. For example, insulin glargine is not currently approved during pregnancy, and only detemir may be used due to the known safety profile during pregnancy [4]. On the other hand, hypoglycemia and poor diabetes control are associated with pregnancy complications and worse neonatal outcomes as well as fetal deformities. The better pharmacokinetic control and the low hypoglycemic rates of insulin degludec compared with other insulins suggest that degludec could be a potential and safe insulin during pregnancy [5]. Degludec use during pregnancy is being investigated in the EXPECT trial (Clinical Trials NCT03377699). In this clinical trial, degludec will be compared to insulin detemir with a primary outcome of last planned HbA1c prior to delivery. Secondary outcomes will evaluate maternal and neonatal safety issues, including preeclampsia, worsening of retinopathy, and hypoglycemia rates. This study began in November 2017, and the expected date of completion will be in 2021. Until the data of the EXPECT trial are available, the only source of clinical evidence of degludec use and safety during pregnancy will be case reports and retrospective studies. To our knowledge, there are only seven published cases including our case using degludec during pregnancy (Table 1) [6-8]

**Table 1:** Table 1. Reported cases of insulin degludec use during pregnancy Table modified from reference [7]. BMI: body mass index, kg: kilograms, g: grams, cm: centimeters, NICU: neonatal intensive care unit, NPDR: non-proliferative diabetic retinopathy, PDR: proliferative diabetic retinopathy.

	Case 1 [7]	Case 2 [7]	Case 3 [7]	Case 4 [6]	Case 5 [6]	Case 6 [8]	Case 7
Mothers							
Age, years	37	26	22	34	22	31	33
Type of diabetes	1	1	1	1	1	2	1
Diabetes duration, years	10	19	13	8	16	3	17
Pre-pregnancy BMI, kg/m^2^	21.5	25	21.6	22.7	36.1	33.9	27
HbA1c (mmol/mol)							
Pre-conception	8.5 (69)	9.4 (79)	9.2 (77)	6.3 (45)	7.7 (61)	6.6 (49)	8.9 (74)
First trimester	7.2 (55)	7.4 (57)	9.9 (85)	6.0 (42)	6.6 (49)	6.6 (49)	6.1 (43)
Second trimester	6.8 (51)	7.3 (56)	7.6 (60)	5.8 (40)	5.7 (39)	5.6 (38)	7.1 (54)
Third trimester	6.7 (50)	6.3 (45)	8.2 (66)	5.2 (33)	5.0 (31)	6.1 (43)	Not done
Micro-/macrovascular complications							
Before pregnancy	NPDR	No	No	No	No	No	PDR
During pregnancy	No	No	No	No	No	No	Pre-eclampsia
Degludec treatment during pregnancy, in weeks	29	5	7	12	8	38	34
Weight gain at the end of pregnancy, kg	6	8	10	9	8	7	14
Time of delivery, weeks	29	35	37	37	37	38	34
Delivery	Caesarean	Caesarean	Caesarean	Caesarean	Induced	Caesarean	Caesarean
Newborn							
Birth weight, g	1730	2900	3930	3330	3300	3280	2500
Length, cm	41	--	50	54.5	50	49	43
Congenital malformations	No	No	No	No	No	No	No
APGAR score at 1 and 5 min	7/10, 8/10	10/10, 10/10	9/10, 10/10	8/10, 9/10	9/10, 10/10	8/10, 9/10	6/10, 8/10
Neonatal hypoglycemia	No	No	No	Yes, mild	Yes, moderate	No	No
NICU admission	Yes (bilirubin increase and episodes of apnea)	No	No	Yes (respiratory distress)	Yes (bilirubin increase)	No	Yes (respiratory distress)

Almost all cases involved type 1 diabetes with variable glycemic control before pregnancy [6-8]. Interestingly, most cases had a delivery with a cesarean section. There were no reported cases of congenital abnormalities. However, 2 newborns had hypoglycemia and more than 50% of the population of newborns required neonatal ICU. These findings may be related to mother diabetic status and related complications. 

## Conclusions

In conclusion, we present the case of a type 1 diabetic patient who was treated during pregnancy with insulin degludec. The use of insulin degludec is not currently approved during pregnancy. Evidence of its safety during pregnancy is limited to case reports, and clearly, the safety and use of this insulin during pregnancy need to be clarified in a randomized clinical trial and in large cohort studies.
